# Mechanical stretch-induced IGF2 overexpression in epidermal keratinocytes promotes hypertrophic scar formation through the IGF1R/p-c-Jun axis

**DOI:** 10.7150/ijbs.91661

**Published:** 2025-01-01

**Authors:** Yuhan Zhu, Lin Chen, Binyu Song, Zhiwei Cui, Guo Chen, Wenjie Dou, Yan Jiao, Juanli Dang, Qing Yang, Zhou Yu, Baoqiang Song

**Affiliations:** Department of Plastic and Reconstructive Surgery, Xijing Hospital, Fourth Military Medical University, Xi'an 710032, China.

**Keywords:** Hypertrophic scar, Mechanical stretch, IGF2, IGF1R, p-c-Jun, Fibroblast, Epidermal cells

## Abstract

Insulin-like growth factor 2 (IGF2) is a mitogenic peptide hormone expressed by various tissues. Although it is three times more abundant in serum than IGF1, its physiological and pathological roles are yet to be fully understood. Previous transcriptome sequencing studies have shown that IGF2 expression is increased in hypertrophic scar (HS); however, its role in HS formation and the underlying mechanism remains elusive. The present study found that IGF2 expression was significantly higher in HS than in normal skin (NS), particularly in epidermal cells. Moreover, mechanical stretch increased IGF2 expression and secretion in keratinocytes, affecting the biological activities of fibroblasts, including proliferation, migration, and collagen synthesis, and transdifferentiated into myofibroblasts after co-culturing keratinocytes with fibroblasts. Mechanistically, keratinocyte-secreted IGF2 activated a nuclear transcription factor phosphorylated c-Jun (p-c-Jun) through insulin-like growth factor 1 receptor (IGF1R) on fibroblast cytomembrane, thereby triggering the profibrotic effect of IGF2. Blocking IGF1R or p-c-Jun inhibited the IGF2-induced profibrotic effect of fibroblasts. Moreover, increased p-c-Jun expression restored the reduction in fibrosis induced by IGF1R knockdown. The IGF2 recombinant protein was also applied to a mouse wound-healing scar model. It was found that IGF2 significantly promoted the formation of HS, whereas IGF2 small molecule inhibitor chromeceptin inhibited HS formation. In conclusion, this study demonstrates that mechanical stretch-induced IGF2 overexpression in epidermal keratinocytes promotes fibroblast activation through the IGF1R/p-c-Jun axis. Therefore, IGF2 may act as a therapeutic target for HS.

## 1. Introduction

Hypertrophic scar (HS) is increasingly becoming a major clinical concern. It often occurs when lesions resulting from lasers, burns and surgical incisions affect the dermis 1. HS is a disease characterized by excessive fibrosis of the skin, usually accompanied by excessive activation of dermal fibroblasts, resulting in the excessive accumulation of extracellular matrix, which thickens the dermis, often raising HS above the skin surface 2. In addition, HS is associated with pain, itching and even limited joint function 3. It is well-established that mechanical stretch is a risk factor for scar formation, which explains the likelihood of HS formation in areas of high skin tension such as joints, anterior chest and lower abdomen 4. Zhao et al. demonstrated that epidermal cells can synthesize and secrete high mobility group box 1 (HMGB1) to promote collagen type I (Col1) and α smooth muscle actin (αSMA) synthesis by dermal fibroblasts, suggesting that the interrelationship between epidermal and dermal cells influences HS formation 5.

Exploring factors influencing HS formation and potential underlying mechanisms has become a research hotspot. Hu et al. found through gene microarray that insulin-like growth factor 2 (IGF2) expression was significantly higher in HS than in normal skin (NS) 6. Our group also found a similar finding in paired human HS and NS samples via transcriptome sequencing. IGF2 is an endogenous secreted protein comprising 67 amino acids 6. It has high structural and functional homology with two other ligands in the insulin family, IGF1 and insulin. IGF2 promotes proliferation and inhibits apoptosis in various cells 7, 8. IGF2 is highly expressed in systemic sclerosis and idiopathic pulmonary fibrosis and promotes extracellular matrix deposition by promoting fibronectin and Col1 expression in lung fibroblasts via phosphoinositide 3-kinase (PI3K) and c-Jun N-terminal kinase (JNK) pathways and disrupting the balance between matrix metalloproteinase 3 (MMP3) and tissue inhibitor of metalloproteinase 1/4 (TIMP1/4) 9, 10. In addition, IGF2 promotes cardiomyocyte hypertrophy through the mammalian target of rapamycin (mTOR) signaling pathway, and its effect is blocked by the insulin-like growth factor 1 receptor (IGF1R) inhibitor 11. IGF2 also promotes the transformation of hepatic stellate cells into myofibroblasts and αSMA expression 12, 13. Using gene microarray, Hu et al. reported that IGF2 expression was significantly higher in HS than in NS 14. However, the mechanism by which IGF2 is increased and its effect on HS are yet to be fully understood.

Phosphorylated c-Jun (p-c-Jun) is a common nuclear transcription factor. Its precursor, c-Jun protein, is involved in the construction of the transcription complex activator protein 1 (AP-1) 15. The transcriptional activity of c-Jun is increased by phosphorylation of serine 63/73 (Ser64/Ser73) in the c-Jun amino-terminal transferase JNK transactivation domain 16. p-c-Jun is implicated in cell proliferation, apoptosis, inflammation and tumorigenesis 17. p-c-Jun is also widely involved in the development of fibrotic diseases. Studies reported that p-c-Jun was enriched on transforming growth factor-beta (TGF-β) promoter in oral epithelial cells after betel nut treatment, thereby promoting transcription of TGF-β and the progression of oral epithelial fibrosis 18. Notably, p-c-Jun was also highly expressed in TGF-β-stimulated cardiac and lung fibroblasts 19, 20. In addition, p-c-Jun binds to the Gα-interacting vesicle-associated protein (GIV) promoter and promotes the expression of GIV. GIV down-regulates caspase-3 levels *in vivo* and is involved in leptin-mediated liver fibrosis 21. Studies have shown that c-Jun regulates the subpopulation of fibroblasts at the trauma site and increases the proportion of reticular fibroblasts, thereby promoting scar formation 22.

Mechanical stretch promotes HS, and tension strips are often used clinically to reduce skin tension around the surgical incision to prevent HS formation 23. IGF2 expression is also correlated with mechanical force. Mechanical shear force-induced IGF2 overexpression promoted MMP12 expression through PI3K signaling pathways in osteosarcoma cells, thus promoting osteosarcoma metastasis and invasion 24. In addition, IGF2 promotes the proliferation of lung fibroblasts and inhibits their apoptosis to increase pulmonary fibrosis 25. IGF2/IGF1R signaling also promoted αSMA expression and contraction capacity in colorectal cancer-associated fibroblasts 26. IGF2 knockdown inhibited Col3 production in TGF-β-stimulated vascular medial smooth muscle cells 27. Previous literature has shown that IGF2/IGF1R can target the transcription factor c-jun 9,28. p-c-Jun, the active form of c-Jun, often forms transcription complexes with other transcription-related factors that promote gene expression. IGF2 promotes the phosphorylation of c-Jun 28. Our RNA sequencing results showed that c-Jun expression in fibroblasts was up-regulated in IGF2-treated groups. It is speculated that IGF2 may promote cell proliferation and migration and extracellular matrix expression through the p-c-Jun transcription factor under the action of mechanical stretch.

The present study found that IGF2 was a key growth factor in promoting HS formation. It also demonstrated that keratinocyte-secreted IGF2 promoted dermal fibroblast proliferation and migration and collagen synthesis and differentiation into myofibroblasts under the action of mechanical stretch. IGF2 bound to the IGF1 receptor on the surface of fibroblasts, activating downstream p-c-Jun transcription factor, thereby increasing collagen and αSMA expression. Moreover, an exogenous IGF2 recombinant protein promoted HS formation in an HS model using total excision of dorsal skin in C57BL/6 mice. The application of IGF2 inhibitor chromeceptin reduced HS formation. Thus, our research suggests that IGF2 could be a target for treating HS.

## 2. Results

### 2.1 IGF2 expression and distribution in human NS and HS

**Figure 1:**
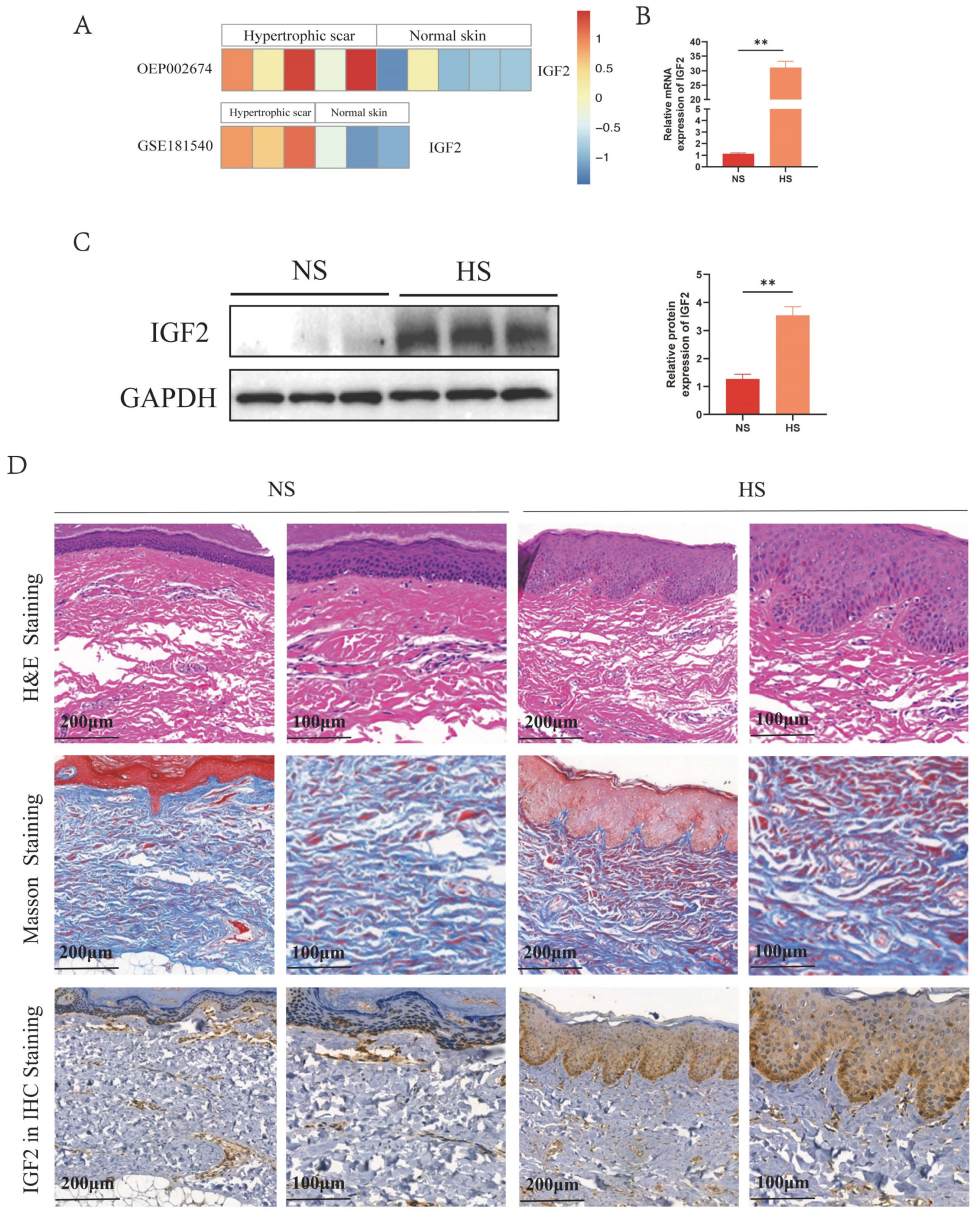
** IGF2 was overexpressed in epidermal cells from the hypertrophic scar.** (A) The expression profile of IGF2 in NS and HS as analyzed in public databases. (B, C) qRT-PCR and WB revealed that the expression of IGF2 in NS and HS. (D) HE, Masson and IGF2 Immunohistochemistry staining of NS and HS. **p < 0.01.

Analysis of public databases revealed that IGF2 was overexpressed in HS compared with NS (Figure 1A). Meanwhile, quantitative real-time polymerase chain reaction (qRT-PCR) and western blot (WB) results showed that IGF2 expression was significantly higher in HS than in NS tissues (Figure 1B, C). In addition, hematoxylin and eosin (HE) staining showed that skin appendages such as hair follicles and sweat glands disappeared in HS compared with NS tissues. Masson staining showed that the dermis of HS contained numerous disorganized collagen fibers compared with NS. Immunohistochemical (IHC) staining showed that IGF2 was overexpressed in HS compared with NS. Moreover, IGF2 was enriched in epidermal cells, especially in basal cells (Figure 1D).

### 2.2 Epidermal cell-secreted IGF2 promotes fibroblast activation

**Figure 2:**
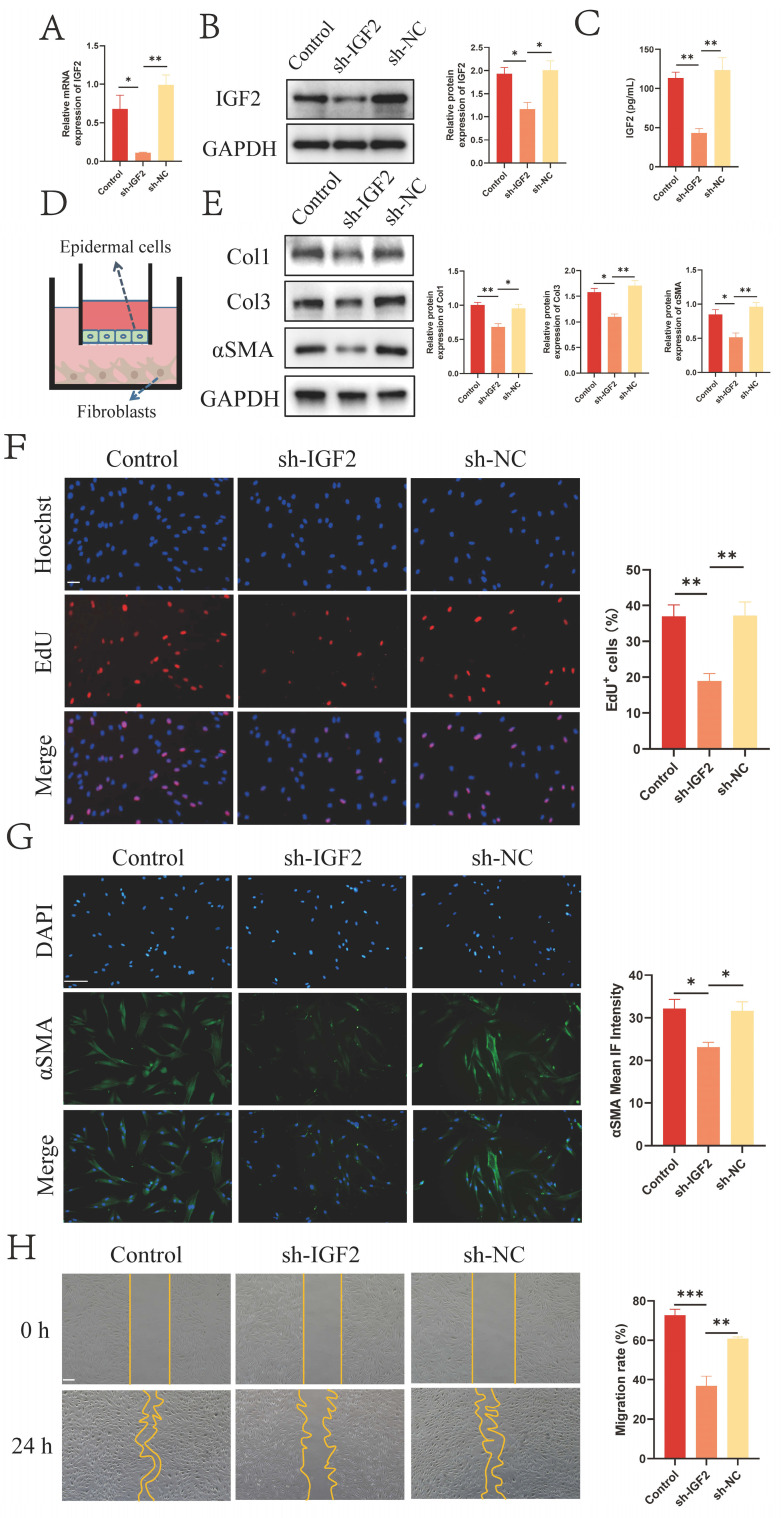
** IGF2 secreted by epidermal cells promotes fibroblasts activation.** (A, B) Downregulated IGF2 expression in HaCaT cells as determined by qRT-PCR and WB. (C) Decreased concentration of IGF2 in the supernatant of HaCaT cells of sh-IGF2 by ELISA. (D) Cell co-culture diagram. (E) Low Col1, Col3, αSMA expression in sh-IGF2 group fibroblasts showed in WB. (F) EdU illustrating the proliferative capacity of Control, sh-IGF2, sh-NC group. (Scale bar=100μm). (G) Cellular immunofluorescence showing the expression of αSMA (Scale bar=100μm). (H) Wound healing assay indicating the migration ability of Control, sh-IGF2, sh-NC group. (Scale bar=100μm). *p < 0.05, **p < 0.01, ***p < 0.005.

IHC staining results revealed that IGF2 was mainly enriched in the epidermis (Figure 1D). IGF2 knockdown and control HaCaT stable transmutation cell lines (sh-IGF2 and sh-NC group) were established by lentivirus infection to investigate the effect of IGF2 synthesized and secreted by epidermal cells on dermal fibroblasts. The knockdown efficiency was verified by qRT-PCR and WB assays (Figure 2A, B). Next, an enzyme-linked immunosorbent assay (ELISA) was utilized to detect the concentration of IGF2 in the supernatant of the sh-IGF2 and sh-NC groups, and the results showed that IGF2 secretion was lower in the sh-IGF2 group than in the sh-NC group (Figure 2C). Then, HaCaT cells from the sh-IGF2 and sh-NC groups were co-cultured with fibroblasts; the co-culture mode is shown in Figure 2D. Meanwhile changes in the biological activity of co-cultured fibroblasts were investigated. WB analysis showed that compared with the sh-NC group, the expressions of Col1, Col3 and αSMA were significantly decreased in the fibroblasts cultured with the sh-IGF2 group (Figure 2E). Immunofluorescence staining also confirmed the decreased αSMA expression in the sh-IGF2 group (Figure 2G). Additionally, 5-ethynyl-2'-deoxyuridine (EdU) and wound healing assays revealed that cell proliferation and migration rates were reduced (Figure 2F, H).

### 2.3 Mechanical stretch promotes IGF2 synthesis in epidermal cells

**Figure 3:**
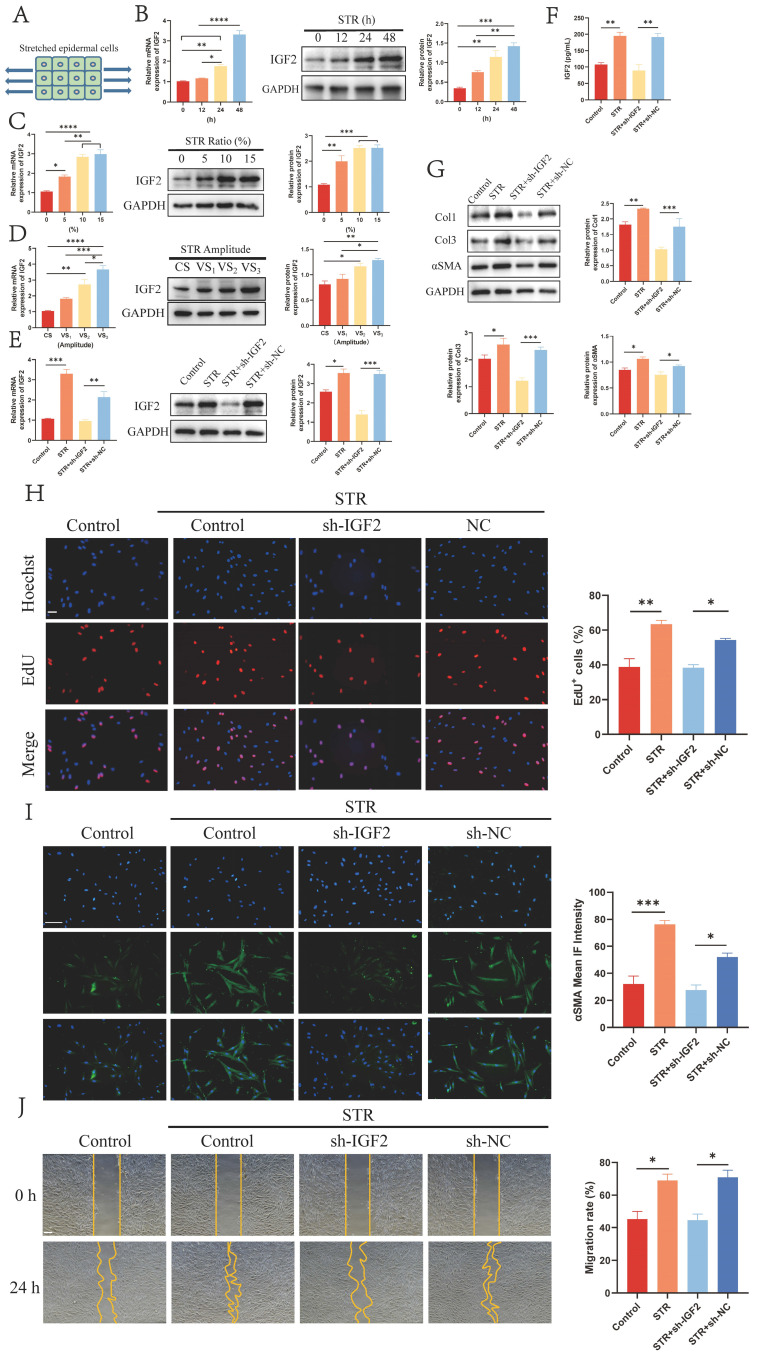
** Mechanical stretch enhanced IGF2 synthesis in epidermal cells.** (A) Schematic illustration of mechanically stretched epidermal cells. (B-D) qRT-PCR and WB analysis of IGF2 expression in HaCaT cells at different stretching times, ratio and amplitude. (E) The IGF2 expression in HaCaT cells of Control, STR (10%, 48h), STR+sh-IGF2 (10%, 48h), STR+NC (10%, 48h) four groups. (F) The concentration of IGF2 in the supernatants of HaCaT cells of Control, STR, STR+sh-IGF2, STR+sh-NC groups. (G) WB showing the protein expression of Col1, Col3, αSMA in fibroblast of four groups. (H-J) The proliferation, αSMA expression and migration ability of fibroblasts in four groups as determined by the EdU, Immunofluorescence and Wound healing assays. (Scale bar=100μm). *p < 0.05, **p < 0.01, ***p < 0.005.

Mechanical stretch plays a vital role in HS formation. Therefore, HaCaT cells were stretched for 0, 12, 24 and 48 h to explore the effect of mechanical stretch on IGF2 expression in epidermal cells. A schematic diagram is shown in Figure 3A. qRT-PCR and WB assays showed that mechanical stretching time- and dose-dependently induced IGF2 overexpression in HaCaT cells (Figure 3B, C). Moreover, constant and variable amplitude was used to stretch HaCaT cells. Results showed that IGF2 expression was upregulated in VS_2_ and VS_3_ cells compared with CS and VS_1_ cells (Figure 3D). These indicated that mechanical stretch induced IGF2 expression in HaCaT cells. Next, Control, STR, sh-IGF2 (STR+sh-IGF2) and sh-NC (STR+sh-NC) HaCaT cells were stretched. qRT-PCR and WB analyses further confirmed that IGF2 expression was increased in the STR group compared with the Control group and in the STR+sh-NC group compared with the STR+sh-IGF2 group (Figure 3E). HacaT cell supernatants of the Control, STR, STR+sh-IGF2 and STR+sh-NC groups were analyzed. Similarly, IGF2 was highly expressed in the STR group compared with the Control group and in the STR+sh-NC group compared with the STR+sh-IGF2 group (Figure 3F). Then, the four groups of HaCaT cells were co-cultured with HS fibroblasts, and results showed that Col1, Col3 and αSMA expression levels and cell proliferation and migration rates were significantly elevated in the STR group than in the control group. Col1, Col3 and αSMA expression levels and cell proliferation and migration rates were significantly decreased in the STR+sh-IGF2 group compared with the STR+sh-NC group (Figure 3G-J). Collectively these data indicate that mechanical stretch promotes IGF2 secretion by epidermal cells, Col1, Col3 and αSMA expression in fibroblasts and cell proliferation and migration ability.

### 2.4 IGF2 activates fibroblasts through IGF1R

**Figure 4:**
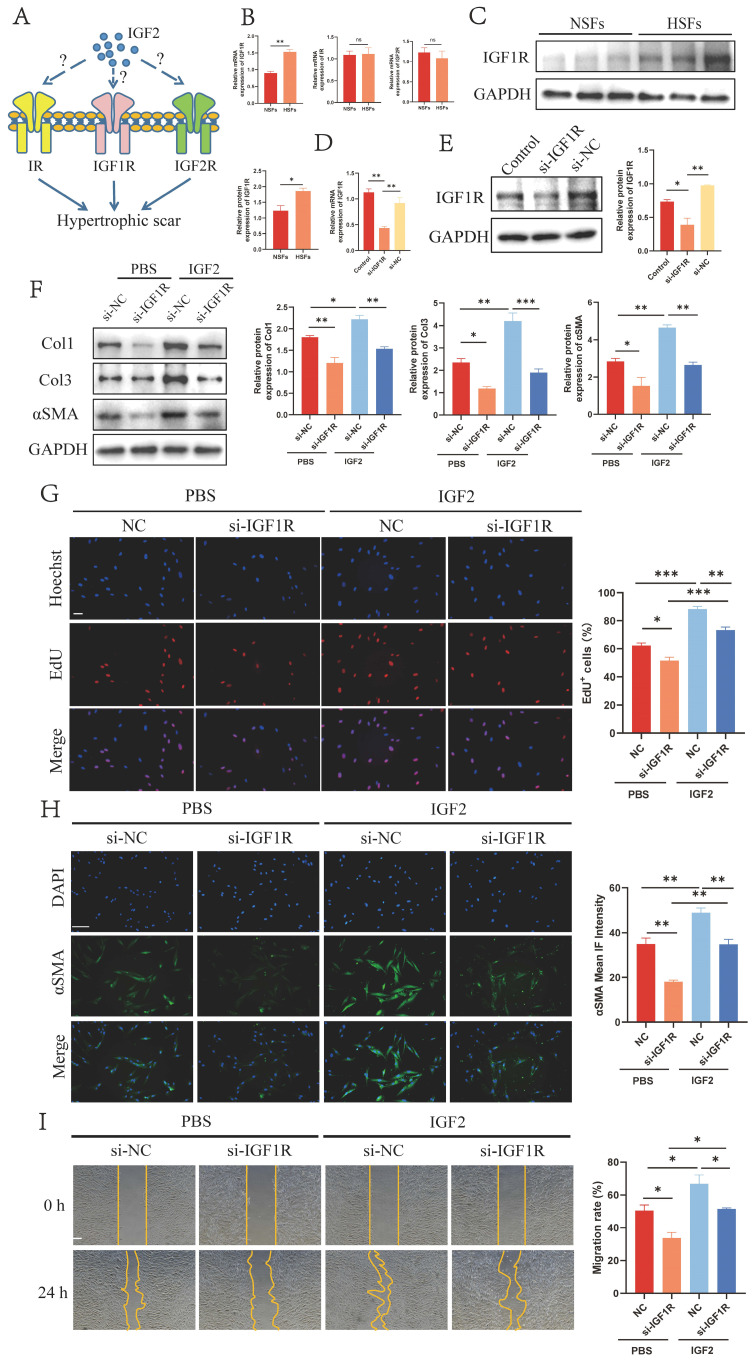
** IGF2 activates fibroblasts through IGF1R.** (A) Schematic of the three receptors for IGF2. (B, C) qRT-PCR and WB analysis of IGF1R expression in NSFs and HSFs. (D, E) qRT-PCR and WB showed decreased si-IGF1R expression compared with si-NC fibroblasts. (F) WB analysis of Col1, Col3 and αSMA expression in PBS+si-NC, PBS+si-IGF1R, IGF2+si-NC and IGF2+si-IGF1R groups. (G) EdU assay showing the proliferative capacity of four fibroblast groups. (Scale bar = 100 μm). (H) Immunofluorescence staining of αSMA expression. (Scale bar = 100 μm). (I) Migratory capacity of four fibroblast groups. (Scale bar = 100 μm). (IGF2: 200 ng/mL). *p < 0.05, **p < 0.01, ***p < 0.005.

Next, we explored IGF2 receptors that promoted HS fibroblast activation. IGF2 bound to three receptors on the surface of fibroblasts IGF1R, insulin receptor (IR) and IGF2R (Figure 4A). IGF2 had the strongest affinity for IGF1R and IR to affect mitogenic function, with IGF2R acting as its elimination receptor. qRT-PCR results revealed increased expression of IGF1R in HS-derived fibroblasts (HSFs) compared to NS-derived fibroblasts (NSFs), whereas IR and IGF2R exhibited no significant expression change between NSFs and HSFs (Figure 4B). Next, WB analysis showed that IGF1R was overexpressed in HSFs at the protein level (Figure 4C).

Subsequently, IGF1R was knocked down by si-IGF1R (si-IGF1R group) in fibroblasts (Figure 4D, E). The expression of Col1, Col3 and αSMA was decreased in si-IGF1R compared with si-NC fibroblasts (Figure 4F, H). Further EdU and migration assays revealed reduced proliferation and migration rates in si-IGF1R fibroblasts (Figure 4G, I). In addition, si-IGF1R and si-NC fibroblasts were stimulated by 200 ng/mL of IGF2 recombinant protein. The expression of Col1, Col3 and αSMA and cell proliferation and migration ability were compared between the phosphate-buffered saline (PBS)+si-NC and IGF2+si-NC groups. Results demonstrated the pro-fibrotic effect of IGF2 on scar fibroblasts. Taken together, these findings suggest that IGF2 promotes fibroblast activation through IGF1R.

### 2.5 p-c-Jun is a key transcription factor mediating IGF2/IGF1R-induced fibroblast activation

**Figure 5:**
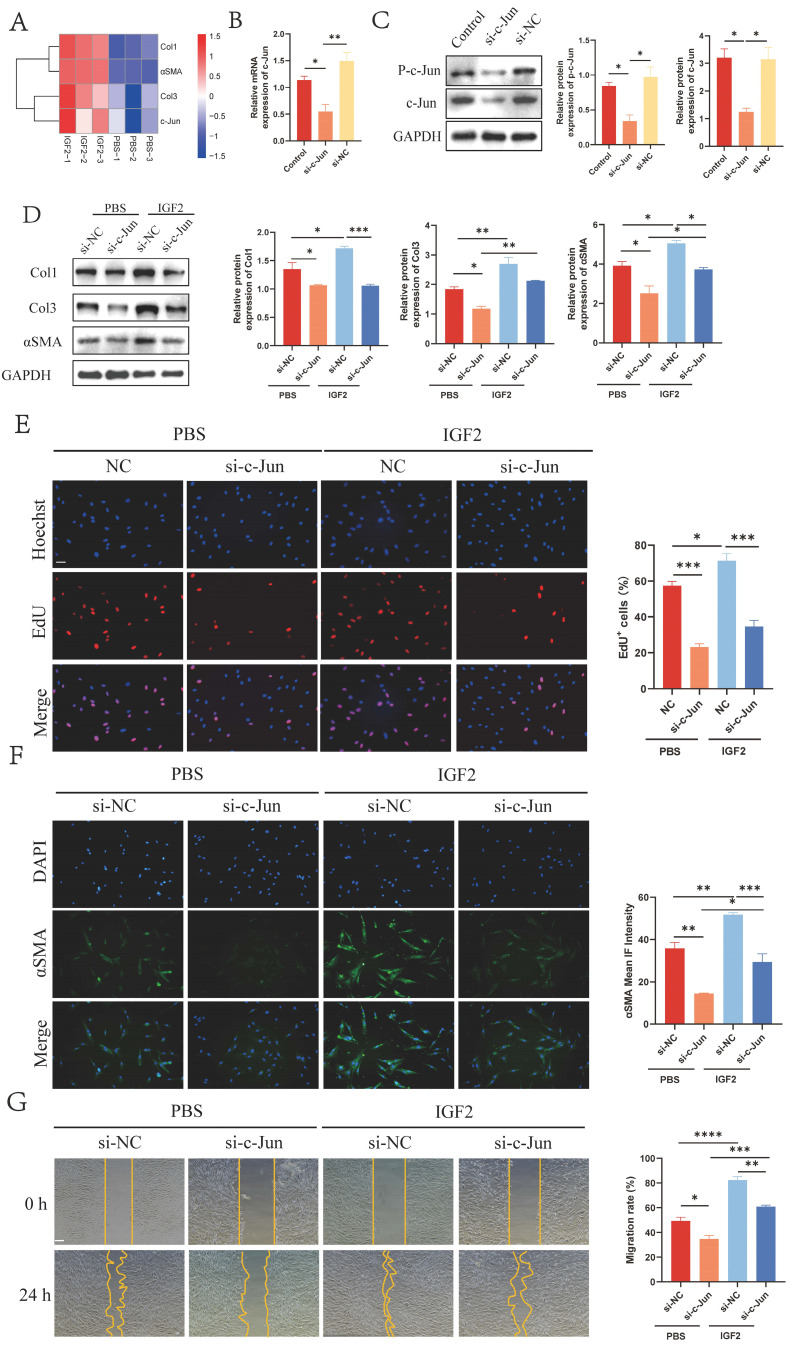
** p-c-Jun is a key transcription factor mediating IGF2-induced fibroblast activation.** (A) RNA-sequencing heatmap showing Col1, Col3, αSMA and c-Jun expression in IGF2 recombinant protein and PBS-stimulated fibroblasts. (B) qRT-PCR analysis of the expression of c-Jun in Control, si-c-Jun and si-NC fibroblasts. (C) WB analysis of the expression and statistics of p-c-Jun in Control, si-c-Jun and si-NC fibroblasts. (D) WB analysis of the expression of Col1, Col3 and αSMA expression in PBS+si-NC, PBS+si-c-Jun, IGF2+si-NC, IGF2+si-c-Jun fibroblasts. (E, G) EdU and wound healing assay showing the proliferative and migratory ability of four fibroblast groups. (Scale bar=100μm). (H) Immunofluorescence staining of αSMA expression. (Scale bar = 100 μm). (IGF2: 200 ng/mL). *p < 0.05, **p < 0.01, ***p < 0.005, ****p < 0.001.

Studies have shown that IGF2 promotes systemic sclerosis-associated pulmonary fibrosis through p-c-Jun 10. Therefore, we determined whether p-c-Jun was the downstream target of IGF2/IGF1R in HS. RNA sequencing (RNA-seq) results showed that c-Jun expression was up-regulated in fibroblasts stimulated by 200 ng/mL of IGF2 recombinant protein compared with the PBS group (Figure 5A). The biological effects of p-c-Jun on HS fibroblasts were also explored. The expression of p-c-Jun by si-c-Jun (si-c-Jun and si-NC group) was reduced in fibroblasts. qRT-PCR and WB analyses confirmed that the expression of c-Jun and p-c-Jun was decreased in fibroblasts compared with the si-NC group (Figure 5B, C). Meanwhile, we further determined whether IGF2 promoted fibroblast activation through p-c-Jun, and 200 ng/mL IGF2 was used to stimulate si-NC (IGF2+si-NC group) and si-c-Jun (IGF2+si-c-Jun group) fibroblasts. WB results showed that the expression of Col1, Col3 and αSMA decreased in the IGF2+si-c-Jun group compared with the IGF2+si-NC group (Figure 5D). Immunofluorescence staining results also showed that αSMA expression was decreased (Figure 5F). EdU and migration assays revealed reduced cell proliferation and migration rates in the IGF2+si-c-Jun than in IGF2+si-NC groups (Figure 5E, G). Overall, these results indicate that IGF2 activates fibroblasts through p-c-Jun.

**Figure 6:**
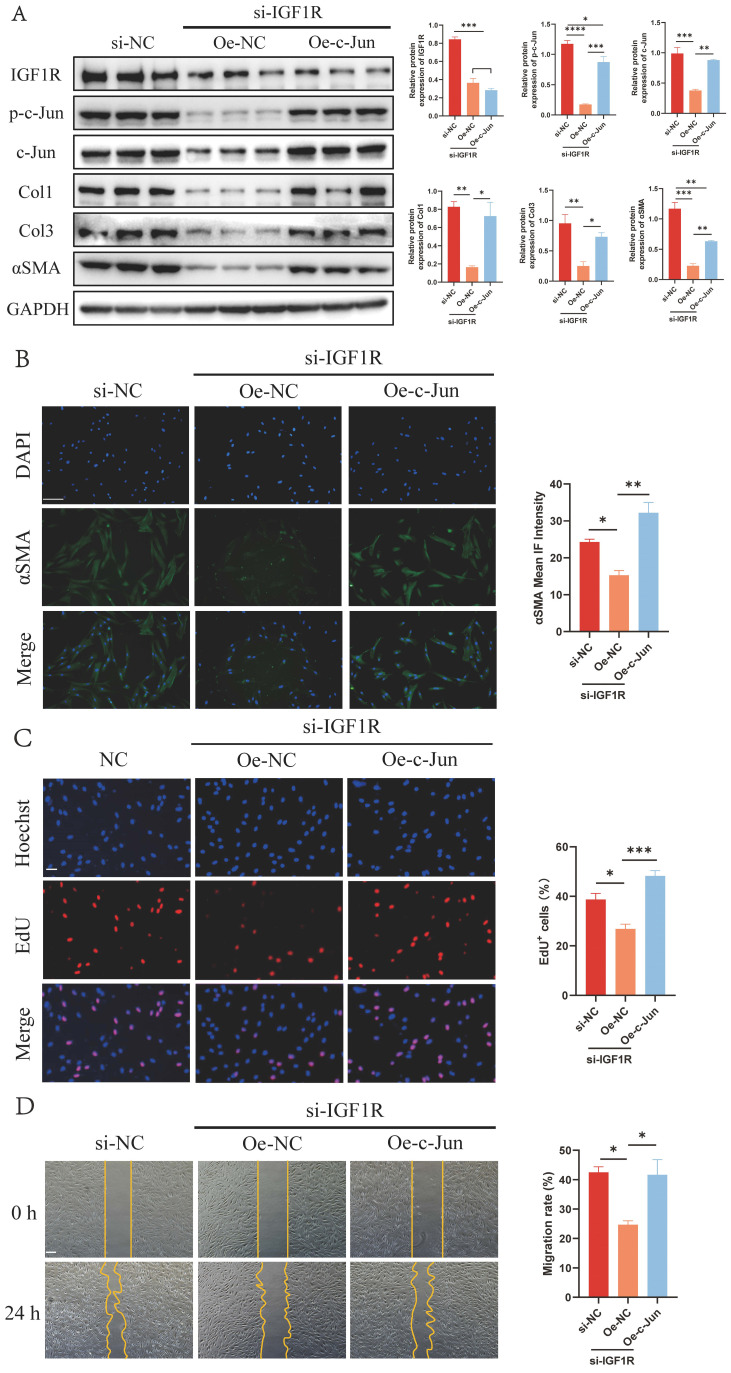
** p-c-Jun is involved in the IGF1R-mediated profibrotic effect in fibroblasts.** (A) WB analysis of the expression levels of IGF1R, p-c-Jun, Col1, Col3 and αSMA. (B) Immunofluorescence staining of αSMA expression. (Scale bar = 100 μm). (C) EdU assay showing the proliferative activity of fibroblasts. (Scale bar = 100 μm). (D) Wound healing assay indicating fibroblast mobility. (Scale bar = 100 μm). *p < 0.05, **p < 0.01, ***p < 0.005, ****p < 0.001.

Furthermore, si-IGF1R fibroblasts were transfected with c-Jun-overexpressed plasmid to determine whether IGF1R was the upstream receptor of p-c-Jun. WB, EdU and wound healing assays confirmed that the increased p-c-Jun expression restored the Col1, Col3 and αSMA expressions and proliferation and migration ability of si-IGF1R fibroblasts to a certain extent (Figure 6A-D). Together, these data imply that IGF2 promotes IGF1R/p-c-Jun-mediated fibroblast activation.

**Figure 7:**
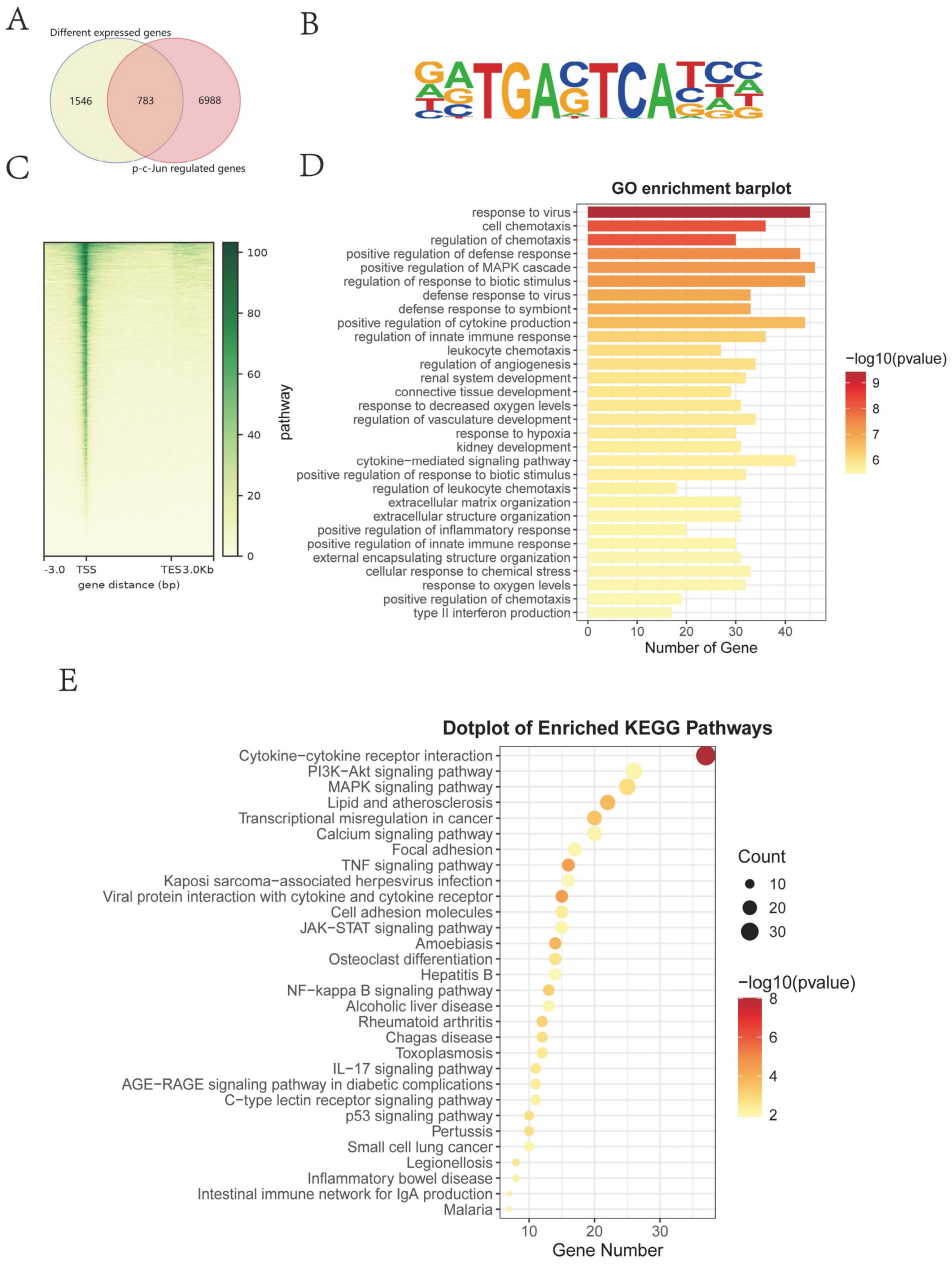
** KEGG and GO enrichment analysis of p-c-Jun regulated genes.** (A) Intersection genes of p-c-Jun regulated genes and DEGs screened from RNA-sequencing in the presence or absence of IGF2. (B) A schematic illustration of the Motif sequence of p-c-Jun. (C) Peak central signal heatmap of the sample. (D, E) KEGG and GO enrichment analysis of p-c-Jun regulated genes in the presence of IGF2.

Next, target genes directly regulated by transcription factor p-c-Jun were screened through the Cleavage Under Targets and Tagmentation (CUT&Tag) assay and intersected with differentially expressed genes (DEGs) from RNA-seq in the presence or absence of IGF2 (Figure 7A). The Motif of transcription factor p-c-Jun is depicted in Figure 7B. Most gene signals were concentrated near the transcription start site (Figure 7C), indicating that the CUT&Tag assay was qualified. The intersected genes were analyzed through the Kyoto Encyclopedia of Genes and Genomes (KEGG) pathway and Gene Ontology (GO) enrichment analyses. These DEGs were enriched in fibrosis-associated pathways such as PI3K/protein kinase B (AKT), mitogen-activated protein kinase (MAPK) and focal adhesion signaling pathways (Figure 7D-E).

### 2.6 IGF2 promotes HS formation in C57BL/6 mice

**Figure 8:**
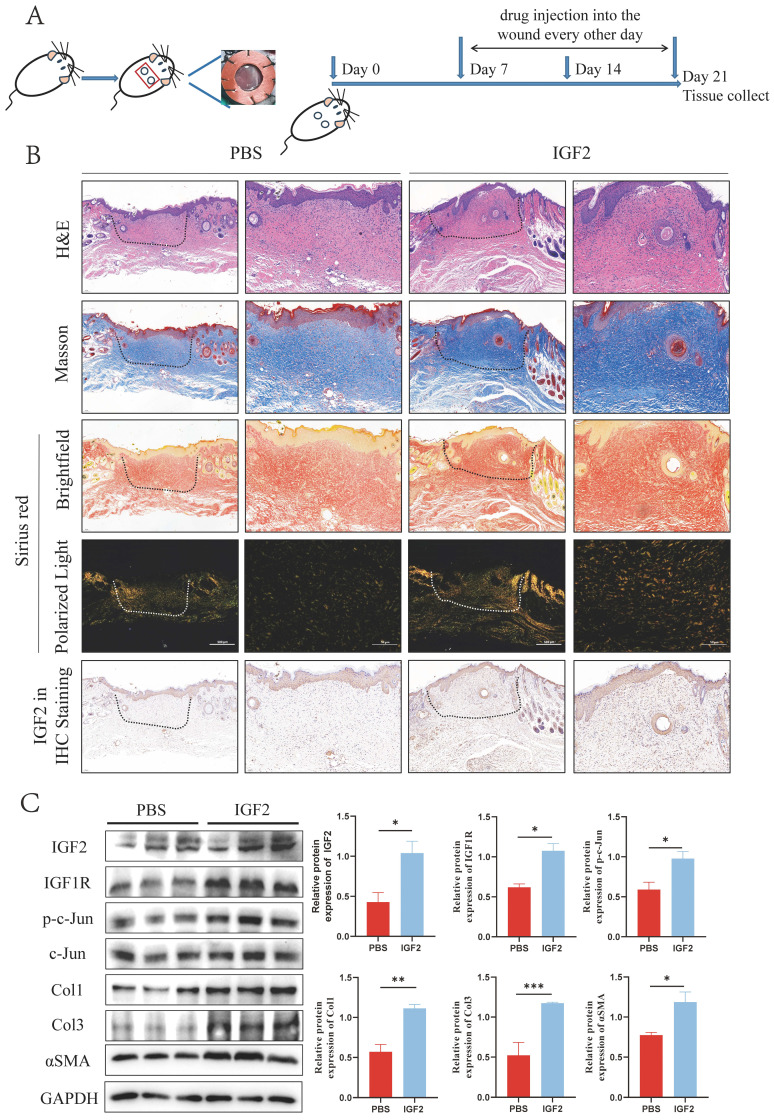

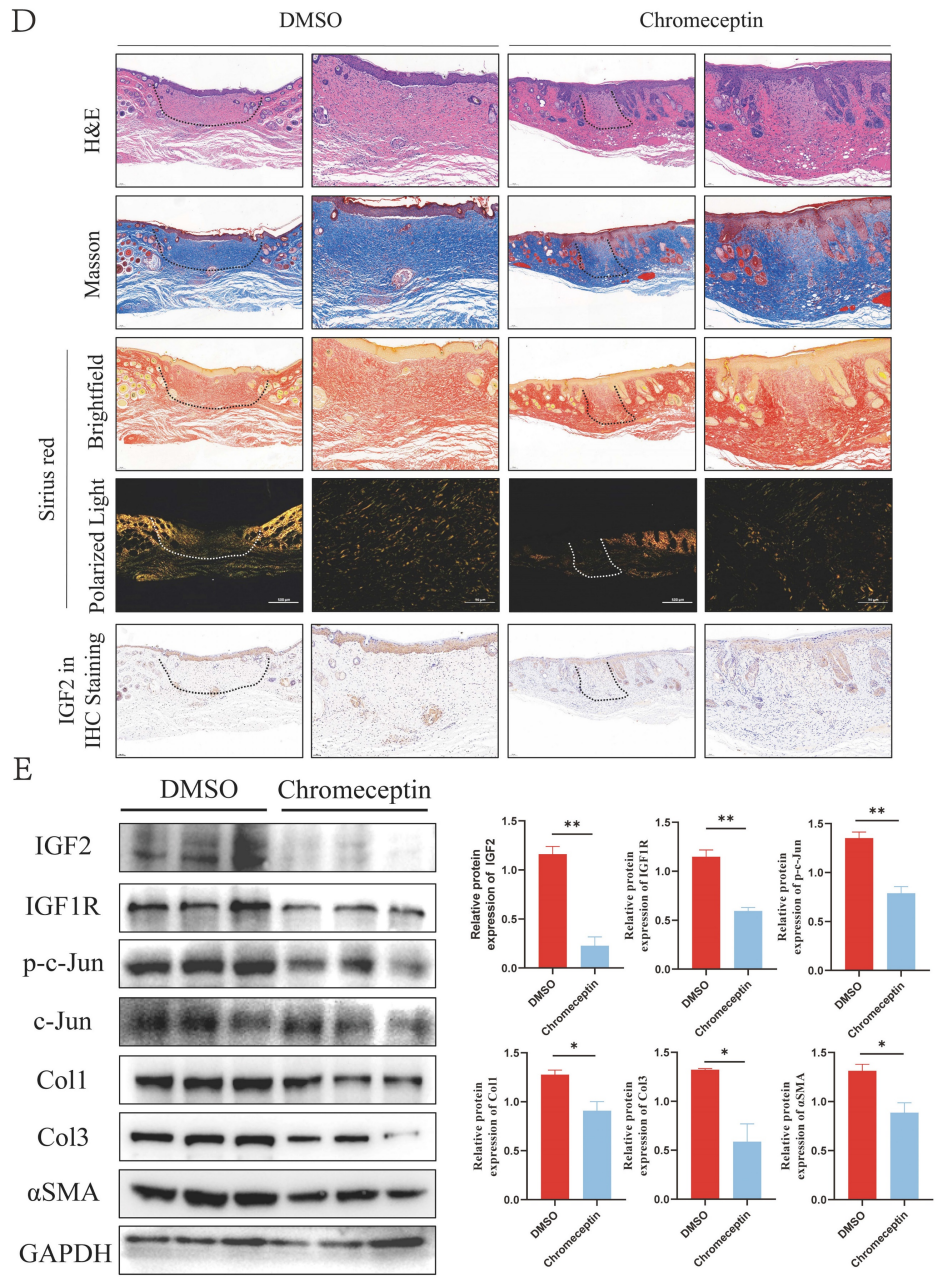
** IGF2 promotes hypertrophic scar formation in C57BL/6 mice.** (A) Schematic diagram of hypertrophic scar model and drug delivery. (B, D) Dermal thickness, collagen synthesis and IGF2 expression determined by hematoxylin and eosin, Masson, Sirius red and immunohistochemical staining. (C, E) WB analysis of protein expression levels of p-c-Jun, Col1, Col3 and αSMA. *p < 0.05, **p < 0.01, ***p < 0.005, ****p < 0.001.

As an inhibitor of IGF2, chromeceptin can inhibit the transcription of IGF2. To study the effects of IGF2 on HS in vivo, 6 mm circular dorsal mouse skin was excised at full thickness and the silicone ring was sutured to create a HS model. Then, from day 7, chromeceptin was injected into the skin wounds daily. Tissue samples were collected on day 21. A schematic diagram of the HS model and drug administration is shown in Figure 8A. HE, Mason and Sirius red staining demonstrated that the IGF2 group exhibited increased dermal thickness, protruding scars, and increased collagen synthesis compared with the PBS group. Compared with the dimethyl sulfoxide (DMSO) group, the chromeceptin group had reduced fibrotic area and collagen synthesis. In addition, IHC staining showed that the expression of IGF2 increased in the whole skin of mice in the IGF2 group compared with that of mice in the PBS group. The expression of IGF2 was decreased in the chromeceptin compared with DMSO groups and was mainly concentrated in the epidermis. Similar to human tissues, the dermis had low expression of IGF2 in mice models (Figure 8B, D). Then, back scar tissues of four groups were collected for protein extraction, and WB analysis revealed that IGF2 promoted collagen synthesis and the expression of IGF1R and p-c-Jun. However, chromeceptin treatment inhibited this effect (Figure 8C, E). Altogether, these findings suggest that IGF2 is critical in promoting HS *in vivo*, and its inhibitor chromeceptin alleviates dermal fibrosis.

## 3. Discussion

HS is the most common skin fibrotic disease, often accompanied by pain, itching and other symptoms. Collagen fiber synthesis and degradation are in a balanced state in NS. Following skin injury, dermal fibroblasts are activated from the static state under the stimulation of inflammation to synthesize large amounts of collagen fibers, resulting in excessive deposition of extracellular matrix, thus promoting the formation of HS. Numerous studies have explored the molecular mechanisms of HS formation, revealing that the TGF-β/ Smad pathway is particularly important in HS. PI3K/AKT pathways also play a role in scar formation. Available treatments for HS are limited and less effective. Therefore, exploring the mechanism underlying HS formation is imperative to identifying potential targets for its treatment.

IGF2, a ligand in the insulin family, is involved in cell proliferation, apoptosis, protein synthesis and other processes. It has been reported that IGF2 is highly expressed in various fibrotic tissues and is involved in the progression of fibrotic diseases. IGF2 promotes fibroblast proliferation and inhibits apoptosis through PI3K/AKT and mitogen-activated extracellular kinase (MEK)/JNK pathways and increases extracellular matrix synthesis to promote pulmonary fibrosis. IGF2 may also be involved in the progression of myocardial fibrosis by disrupting the TIMP2/MMP9 balance. IGF2 is highly expressed in several fibrotic animal models, such as hypoglycemia-induced right ventricular fibrosis, carbon tetrachloride-induced liver fibrosis and unilateral ureteral obstruction-induced renal fibrosis. It has been reported that the expression of IGF2 is also significantly increased in HS; however, the molecular mechanism by which IGF2 promotes fibrosis remains elusive. WB and Masson staining showed that IGF2 expression was increased in HS and enriched in the epidermis. Tension strips are often used to reduce skin tension and prevent HS formation clinically. The principle of action is to resist the force of the wound extending from the surface of the skin into the depth 29. Therefore, although fibroblasts in the lower dermis are the main effector cells in HS, studying the effects of mechanical stretch on epidermal cells in the upper epidermal layer and the interaction between epidermal cells and fibroblasts is paramount. This has a certain guiding significance for reducing HS formation. In addition, immunohistochemical results showed that IGF2 was mainly expressed in epidermal cells, especially in basal cells. Therefore, it is particularly important to focus on the effects of IGF2 synthesis and secretion in epidermal cells on the biological activities of fibroblasts. Epidermal cells were co-cultured with scar fibroblasts, and the results of WB, immunofluorescence staining, EdU, and scratch experiments confirmed that IGF2 secreted by epidermal cells promoted the expression of fibroblast fibrotic proteins and proliferation and migration rates.

Mechanical stretch is the most common risk factor for HS formation, which is more likely to occur in areas of skin tension. Mechanical stretch is usually time- and strength-dependent on the activity of HS fibroblasts. He et al. found that mechanical stretch promoted the influx of cellular calcium ions by activating the expression of piezo-type mechanosensitive ion channel component 1 (Piezo1), a cationic channel in fibroblasts, thereby promoting the proliferation, migration and contraction of fibroblasts 30. Recent studies have shown that mechanical stiffness promotes the proliferation of fibroblasts and inhibits their apoptosis by forming a positive feedback loop of Piezo1-Wnt2/Wnt11-C C motif chemokine ligand 24 (CCL24), which prevents hypertrophic scar formation. Blocking Piezo1 expression mitigated the formation of HS 31. The formation of HS is complicated by mechanical forces. Besides fibroblasts, mechanical stretch also promotes calcium flow in epidermal cells 32. Various skin cells, such as Langerhans cells and melanocytes, also have ion channel receptors on their surfaces, which convert mechanical signals into biological signals. In addition, multiple signaling pathways, such as the inflammatory focal adhesion kinase (FAK)-extracellular-related kinase (ERK)-monocyte chemoattractant protein 1 (MCP-1) and integrin-β1-p130 Crk-associated substrate (P130Cas) and p38MAPK pathways, are involved in the development of HS 33. External mechanical stretch may be induced differently by joint activity, and the biomechanics inside NS and HS are also different. NS is soft and elastic, whereas HS is stiff. The proper proportion of Col1 and Col3 and elastin in the NS and the woven network structure make the skin have favorable elasticity, hardness and deformation ability. The disproportional and disordered distribution of the extracellular matrix in HS causes the skin to lose these properties 34. Some studies have endeavored to prevent the formation of HS by reducing mechanical stretch. Zhang et al. inhibited fibrotic protein expression such as collagen and TGF-β1 in fibroblasts through a microneedle patch and alleviated the degree of HS in rabbit ears 35. The present study found a time- and dose-dependent increase in IGF2 synthesis in epidermal cells.

Numerous mechanical signaling pathways, such as integrins/FAK, Wnt/β-catenin, Yes-associated protein (YAP)/transcriptional coactivator with PDZ-binding motif (TAZ) and PI3K/AKT signaling pathways, are involved in the mechanical stretch transduction of fibroblasts, thereby activating cellular responses 23. Integrins are heterodimers that connect the extracellular matrix with the intracellular skeleton. Within the cells, integrins are bound to the intracellular skeleton by binding to the F-actin proteins. This multifunctional adaptor molecule structure is called the talin protein 36. FAK is a non-receptor intracellular tyrosine kinase whose phosphorylation activity is significantly up-regulated in HS and scar fibroblasts and is mainly involved in the transduction of local adhesion signals 37. The extracellular matrix of NS is soft and the talin protein structure is closed. The conformation of talin protein changes with an increase in the mechanical stretch and stiffness of the cells and then the local adhesion factors are recruited, promoting the proliferation, contraction and collagen synthesis of fibroblasts 38. The Wnt/β-catenin pathway is actively expressed in tissues with high cell renewal. The structure of the destruction complex (DC) regulates the activity of β-catenin. The DC encompasses axin, glycogen synthase kinase 3, adenomatous large intestine polyposis (APC), protein phosphatase 2A (PP2A), casein kinase 1 (CK1) and E3-ubiquitin ligase transducin repeat-containing protein (TrCP) 39. When the Wnt signal is not activated, the DC binds to β-catenin and mediates its degradation. The activation of the Wnt signal by mechanical stretch promotes the binding of Wnt to LRP and Frizzled receptors on the cell membrane, recruiting DC component proteins axin and disheveled to the cell membrane. These structural changes in the DC inhibit the degradation of β-catenin. Then β-catenin enters the nucleus and promotes the transcription of pro-fibrotic genes, such as TGF-β 40. YAP and TAZ are up-regulated in keloid fibroblasts. When mechanical stretch increases, YAP/TAZ transfers to the nucleus and binds to DNA-binding transcription factors (TEADs) to promote gene transcription and ultimately cell proliferation. Reduction in YAP/TAZ expression by siRNA inhibits the migration, proliferation and collagen synthesis of fibroblasts 41. The PI3K/AKT signaling pathway plays a crucial role in cell biological processes such as proliferation, migration and metabolism. A previous study found that fibroblasts from a mechanically drawn site of the mice had up-regulated phosphorylated AKT expression compared with fibroblasts from a non-mechanically drawn site 42. PI3K/AKT signal may be involved in TGF-β-mediated promotion of αSMA expression in fibroblasts, and inhibition of its activity may reduce the proliferation, migration and synthesis of TGF-β in fibroblasts 43. In addition to the aforementioned mechanical stretch-sensitive signaling pathways, some cation channels, such as Piezo proteins and transient receptor potential (TRP) channels, are also involved in the transduction of cellular mechanical signals, which are both involved in mechanical stretch-induced calcium inflow 23. TRPC is the most important ion channel in the TRP family, and TRPC6-mediated calcium inflow promotes the differentiation of fibroblasts into myofibroblasts 44. Piezo1 is significantly up-regulated in scar fibroblasts, and both mechanical stretch and stiffness promote its expression. The positive feedback loop of Piezo1-Wnt2/11-CCL24 in skin fibrosis facilitates the increase in the stiffness of the skin extracellular matrix, thus further promoting skin fibrosis 31. According to previous studies, various effector factors are involved in mechanical stretch to promote HS. The current study found that mechanical stretch promoted the expression of IGF2 in epidermal cells as well as the proliferation and migration ability and collagen synthesis of fibroblasts through the IGF1R/p-c-Jun axis. This may provide a target for preventing HS. However, it mainly focuses on the expression and mechanism of IGF2 in HS and does not explore other mechanical sensitivity factors. Therefore, whether IGF2 has a greater effect on HS than other effector factors remains unclear. Our future studies will explore the effects of different mechanical sensitivity factors alone or in combination on HS.

Due to the limitations of the mechanical stretch instrument, we only used unidirectional mechanical stretch. The stretch directions of different parts of the wound during the healing process were different. This shows that although the present study followed the most common cell stretching model, there are still limitations to stretch cells *in vivo*. Recently, cell-matrix adhesives with different mechanical stiffness have been prepared using collagen-coated polyacrylamide hydrogels 31. This indicates that the study of mechanical stretch for HS has been extended from two to three dimensions. Previous studies also found that mechanical stiffness promoted Piezo1 to regulate the metabolism of arginine and proline in fibroblasts, thereby promoting collagen production and synthesis 45.

The frequently used animal models of hypertrophic scar include rabbit ear scar model, rat tail scar model and Duroc pig model. The procurement and rearing costs of rabbits are low and they have a strong anti-infection capacity, but their wounds often occur in the perichondrium of the ear. Scars generated in Duroc pigs are mainly similar in terms of skin thickness and pathological features to the hypertrophic scar in humans, but they are expensive and excessively large. Unlike in the human skin, mice wounds can easily shrink. To investigate back wound models in mice, researchers commonly employ mechanical pull devices, which are prone to detachment. To address this limitation, researchers initially utilized a hole punch to create a 6mm circular wound on the backs of mice, followed by suturing the wound with a silicone ring to normal skin. This approach prevents rapid wound contraction and facilitates the formation of hypertrophic scar. Silicone rings are soft and light, so the rings are not easy to fall off and do not restrict the activity of mice 22. The result revealed that IGF2 promoted hypertrophic scar formation *in vivo*, while the IGF2 inhibitor (Chromeceptin) prevented the formation of hypertrophic scar.

As a transcription factor, p-c-Jun modulates the progression of fibrotic diseases. It has been demonstrated that p-c-Jun is enriched in the promoter region of TGF-β in oral epithelial cells and promotes its transcription. p-c-Jun is the most important downstream transcription factor of the JNK pathway. Furthermore, IGF2 promotes lung fibroblast proliferation and collagen synthesis through the JNK pathway. In our study, we initially identified IGF1R and p-c-Jun as key mediators in the process of IGF2 promoting scar fibroblast activation, utilizing small interfering RNAs to investigate their role. Subsequently, the response test demonstrated that the increase of p-c-Jun expression restored the inhibitory effect of si-IGF1R on fibroblast activation. In summary, this study indicated that IGF2 activated the transcription factor p-c-Jun via IGF1R on the fibroblast surface, thereby promoting fibroblast proliferation, migration, and collagen and αSMA synthesis.

## 4. Materials and Methods

### 4.1 Skin specimen collection and Human ethics

Tissues samples of normal skin and hypertrophic scar were collected from Department of Plastic and Reconstructive Surgery, Xijing Hospital, Fourth Military Medical University. All patients provide informed consent forms to participate in the study. The study was approved by the Ethics Committee of Xijing Plastic Surgery Hospital, ethics number for KY20242186-C-1. Skin tissues were collected for RNA and protein extraction, followed by primary fibroblasts culture and chemical staining.

### 4.2 Real-time Quantitative PCR (qRT-PCR)

Total RNA was extracted by TRIzol and reverse-transcribed to cDNA with the reverse transcription reagent. The cDNA was subjected to qRT-PCR using the following procedure: predenaturation at 95°C for 5 min, 40cycles of denaturation at 95°C for 10s, annealing and extension at 60°C for 30 s in line with the instructions of the rapid procedure (Yeasen Biotechnology, Shanghai, China). The mRNA expression levels of target genes were normalized to that of GAPDH. The following primers were used. Human GAPDH: Forward 5'-ACAACTTTGGTATCGTGGAAGG-3' and Reverse 5'-GCCATCACGCCACAGTTTC-3', Human IGF2: Forward 5'-GTGGCATCGTTGAGGAGTG-3' and Reverse 5'- CACGTCCCTCTCGGACTTG-3', Human IGF1R: Forward 5'-AGGATATTGGGCTTTACAACCTG-3' and Reverse 5'-GAGGTAACAGAGGTCAGCATTTT-3', Human IGF2R: Forward 5'-CTGCCGCTATGAAATTGAGTGG-3' and Reverse 5'-CGCCGCTCAGAGAACAAGTT-3', Human IR: Forward 5'-CGGCCTCTACAACCTGATGAA-3' and Reverse 5'-TACGGGACCAGTCGATAGTGG-3', Human c-Jun: Forward 5'-TCCAAGTGCCGAAAAAGGAAG-3' and Reverse 5'-CGAGTTCTGAGCTTTCAAGGT-3'.

### 4.3 Western Blotting (WB)

Tissue or cell proteins are extracted by treatment with RIPA containing the protease inhibitor and phosphatase inhibitor on ice. The protein concentration was determined using the BCA protein assay kit (Beyotime, China). Equal amounts of the proteins were electrophoreted on 7.5%, 10% SDS-PAGE gels and transferred to PVDF membrane. Membrane was blocked with 5% skim milk and incubated with primary antibody at 4°C for 12 hours. Subsequently, it was incubated with HRP-IgG secondary antibody at room temperature for 2 hours. Protein bands were visualized using the SuperSignal^TM^ West Atto (Thermo Fisher Scientific, USA), and expression bands were analyzed by Image J software. The protein expression levels were normalized to the expression of GAPDH. The primary antibodies used were: GAPDH (1:5000, Proteintech, China), IGF2 (1:2000, ABclonal, China), Col1 (1:1000, Proteintech, China), Col3 (1:1000, Proteintech, China), αSMA (1:1000, Proteintech, China), IGF1R (1:1000, CST, USA), p-c-Jun (1:1000, CST, USA), c-Jun (1:1000, HUABIO, China).

### 4.4 Hematoxylin and Eosin staining (HE), Masson's Trichrome staining (Masson), Sirius Red staining and Immunohistochemistry (IHC)

Tissues were fixed in 4% paraformaldehyde and used to prepare paraffin embedded sections, which were dyed with Hematoxylin and Eosin, Masson and Picrosirius red. In Sirian red staining, Col1 appears red, while Col3 appears green under polarized light. For immunohistochemistry, the tissues were incubated with primary antibody at 4°C overnight, followed by incubation with the enzyme linked secondary antibody at 37°C for 30 min, and the nuclei were stained with DAB and hematoxylin. Primary antibody used was IGF2 (1:400, ABclonal, China).

### 4.5 Isolation and Culture of Normal skin and Hypertrophic scar fibroblasts (NSFs and HSFs)

Briefly, epidermis and subcutaneous fat of skin tissue were removed and remaining dermis was cut into 2×2mm pieces and spread in T25 culture bottle. The sections were emersed in DMEM enriched with 10% FBS and 1%penicillin-streptomycin, which was changed every three days. Fibroblasts usually started to crawl out on day 14 and could be passaged when fusing to 80-90%.

### 4.6 Stable short hairpin RNA-IGF2 (sh-IGF2) HaCaT cells construction

Lentiviral suspension was collected from 293T cell supernatant by a lentivirus package system by pLP1, pLP2, pVSV-G and the destination plasmid (PTSB-SHIGF2-copGFP-2A-PURO) (Tsingke Biotechnology Co., Ltd., China). HaCaT cells were cultured in six-well plates until they reached a confluence of 70%, the cells were infected with lentivirus for 24 h and replaced with fresh medium. Stable transmutation strains were screened with 1μg/mL of purinomycin for 24 h. The target of sh-IGF2 was 5'-CGCTCAGAAACCAAATTAAAC-3'.

### 4.7 HaCaT cells mechanical stretching

The HaCaT cells were stretched using the Mechanical Cell Strain Instrument (NST-1400-04). 2μg/cm^2^ rat tail tendon collagen type 1 (Solarbio, C8065) was coated in chambers with a thin layer and dried at room temperature for 2 h. The chambers were washed with PBS and the HaCaT cells were added to the chambers with complete medium. The cells were stretched after 24 h. Stretching ratio: 0%, 5%, 10%, 15%, 20% for a stretching distance of 0mm, 1mm, 2mm, 3mm and 4mm, respectively. Stretching amplitude were as follows. CS: Constant stretching amplitude, Ratio at 5%, 48h). VS: Variable stretching amplitude. VS_1-3_: Ratio at 5% for 4 h and 10/15/20 % for 4 h, then repeat until 48 h.

### 4.8 Enzyme-Linked Immunosorbent (ELISA) assay

The concentration of IGF2 in the HaCaT culture supernatant was measured using the Valukine^TM^ ELISA kit (Novus, USA), and the specific procedures were performed following the instructions. Absorbance at 450 nm was quantified and the concentration of IGF2 was calculated from a standard curve within the effective range.

### 4.9 EdU cell proliferation assay

The HSFs cells were cultured in 24-well plate until they reached 70% confluence. The cell medium containing EdU at a concentration of 10mM was added, incubated for 12h. Next, the cell medium was discarded, fixed with 4% paraformaldehyde, washed with PBS containing 3% BSA, and permeated with PBS containing 0.3% Triton X-100. The Click reaction solution was prepared following the instructions on the kit (C0078S, Beyotime), and was added to the 24-well plate and incubated for 30 min in the dark. The nuclei were then stained with 1X Hoechst at room temperature for 10 min. Cell fluorescence detection was conducted using a microscope. The Nuclei were stained blue, EDU-labeled cells appeared red, and the proportion of EDU-labeled cells in all cells was used as the indicator to detect cell proliferation activity.

### 4.10 Wound healing assay

The HSFs were cultured in a six-well plate until they fused to 100 %. A vertical line was drawn at the bottom of the six-well plate using a 200μL pipette tip. Excess cells were washed from the scratched area with PBS. Scratched area was analyzed by Image J software at 0 h and 24 h. HSFs migration rate was equal to 1- Scratched area at 24 h/ Scratched area at 0 h.

### 4.11 Immunofluorescence (IF)

The HSFs were fixed with 4% paraformaldehyde and then permeated with 0.5%Triton X-100. Subsequently, the cells were blocked with 2%BSA and then incubated with primary antibody (αSMA, 1:400, Proteintech, China) overnight at 4°C. The next day, they were fixed with the goat anti-rabbit IgG (H+L) Alexa Fluor 488 second antibody (1:2000, Thermo Fisher Scientific, USA) for 30 min and the nuclei were stained with DAPI (1:5000, Beyotime, China).

### 4.12 Transfection with siRNA and overexpressed plasmid

siRNA and overexpressed plasmid were transfected into HSFs using the Lipofectamine^TM^ 3000 reagent following the manufacturer's instructions (Thermo Fisher Scientific, USA). The siRNA sequences used were: si-IGF1R: 5'-CAAUGAGUACAACUACCGCUU-3', si-c-Jun: 5'-AGTCATGAACCACGTTAAC-3' (Tsingke Biotechnology Co., Ltd., China). Overexpressed plasmid of c-Jun was purchased from MIAOLING BIOLOGY, China (P16701).

### 4.13 RNA-sequencing

Total RNA was extracted from cells using the TRIzol reagent. The RNA concentration was determined with NanoDrop2000. It was then segmented and purified by Oligo(dT)-attached magnetic beads. Next, it was used to synthesize cDNA first chain and second chain, followed by repair of the end of cDNA, addition of RNA index adapters and purification of the products. After PCR amplification, the PCR products were purified by Ampure XP Beads and the quality was validated and controlled by Agilent Technologies 2100 bioanalyzer. Subsequently, single chain circular DNA was synthesized from double chain stranded cDNA products and used to construct the final library. DNA nanoballs were prepared by amplifying the final library through MGI2000 platform (Tsingke Biotechnology, China). Raw data were generated through base calling using MGISEQ-2000, followed by quantitative gene expression analysis through comparison of the raw data with the reference sequence. Differentially expressed genes (DEGs) in the two groups were identified using the R package DESeq2, with a significance threshold set at an adjusted p-value < 0.05 and |Log2-fold change (FC) | ≥ 1.

### 4.14 Cleavage Under Targets and Tagmentation (CUT&Tag) assay

The CUT&Tag seq was performed by the Romics Biotechnology Ltd (Shanghai, China). Briefly, after pre-treatment of HSFs, DNA was extracted and sequenced by high throughput by staining with primary antibody that bind to the target protein, secondary antibody that bind to the primary antibody. It was then activated with transposase and the DNA was fragmented using the pA-TN5 enzyme binding to the antibody. CHIP level p-c-Jun (#3270, CST, USA) primary rabbit antibody was used to screen for genes bound to the motif of p-c-Jun transcription factor. The intersection of the genes regulated by p-c-Jun transcription factor and differentially expressed genes (DEGs) in the RNA-seq data which were visualized through a Venn diagram. These genes were analyzed by Kyoto Encyclopedia of Genes and Genomes (KEGG) and Gene Ontology (GO) analysis.

### 4.15 Animal models and Animal ethics

A 6mm wound was created on the back of C57BL/6 mice with a hole punch, and then sewed a silicone ring with an inner diameter of 6mm to the outer edge of the wound to prevent wound contraction. On day 7-21, PBS, 15μg/Kg/d IGF2 (P09535, CUSABIO, China) dissolved in PBS or DMSO, 1.5mg/Kg/d Chromeceptin (HY-115449, MCE, USA) dissolved in DMSO) were injected at the wounds every other day. On day 21, the scar tissues on the back of C57BL/6 mice and the surrounding normal skins were collected for HE, Masson and Sirus red staining. All experimental protocols were approved by the animal ethics center of the school, Fourth Military Medical University, ethics number for IACUC-20220571.

### 4.16 Statistical analysis

All experiments were repeated three times, and the results were counted using Graphpad Prism. The data were expressed as the mean ± standard deviation. The data were analyzed using student t test and one-way analysis of variance.

## Figures and Tables

**Figure 9:**
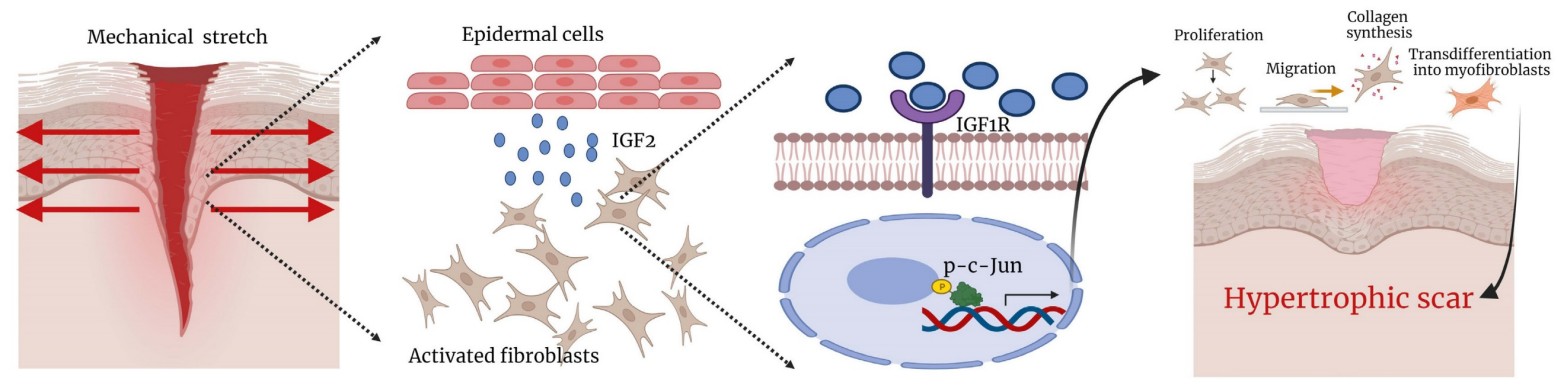
Summary diagram illustrating the molecular mechanism.

## Abbreviations

IGF2: insulin like growth factor 2
IGF1R: insulin like growth factor 1 receptor
HS: hypertrophic scar
NS: normal skin
Col1: collagen type I
Col3: collagen type III
αSMA: α smooth muscle actin
qRT-PCR: real-time quantitative polymerase chain reaction
WB: western blotting
HE: hematoxylin and eosin staining
IHC: immunohistochemistry
CUT&Tag: cleavage under targets and tagmentation
